# Custom-made Trabecular Metal Acetabular Component in Total Hip Revision

**DOI:** 10.1055/s-0041-1735142

**Published:** 2022-09-05

**Authors:** Roberto Dantas Queiroz, David Jeronimo Peres Fingerhut, Luiz Henrique Saito

**Affiliations:** 1 Serviço de Ortopedia e Traumatologia, Hospital do Servidor Público Estadual de São Paulo, São Paulo, SP, Brasil; 2 Serviço de Ortopedia e Traumatologia, Santa Casa de Misericórdia de Marília, São Paulo, SP, Brasil; 3 Serviço de Ortopedia e Traumatologia, Irmandade Santa Casa de Londrina – ISCAL, Londrina, PR, Brasil

**Keywords:** 3D printing, acetabulum, arthroplasty, replacement, hip, hip prosthesis, titanium

## Abstract

The following case report aims to demonstrate a total hip arthroplasty revision surgery (THARS) using a custom-made trabecular metal acetabular component for correction of a severe acetabular defect. Currently, in the literature, there are few complete descriptions of surgical planning and procedures involving customized prostheses. This is due to the inherent technical difficulty of the surgical procedure and the high costs related to the planning and materials.

## Introduction

In the last decades, several studies have been brought to light that show new techniques and materials to find reasonable and feasible solutions to correct hip bone defects, with the goal of allowing patients to restore sufficient joint function. In the current study, we present the use of a custom-made trabecular metal revision acetabular prosthesis made of titanium in a patient with Paprosky type III-B classification of acetabular bone loss. 1

## Case Report

This is the case of a 65-year-old Caucasian female patient with a history of degenerative disease of the right hip, presenting pain and decreased range of motion. She underwent total right hip arthroplasty at the age of 30, using a cemented total prosthesis, without any complications. After 15 years, she presented discomfort and limited movement of the right limb, undergoing a new surgical procedure for revision of the acetabular component using an acetabular reinforcement ring. Nineteen years after this procedure, the patient experienced discomfort and severe loss of range of motion again. After assessing the results of her imaging studies, it was evidenced the loosening of the prosthetic components in the acetabulum and concomitant acetabular bone loss, which was classified as Paprosky type III B, and a new approach was indicated.

Serial evaluations up to the 6 ^th^ month after the surgery revealed a rapid gain in the range of motion, osseointegration of components, and patient satisfaction with the result.

## Steps in customizing the acetabular component

**Fig. 1:**
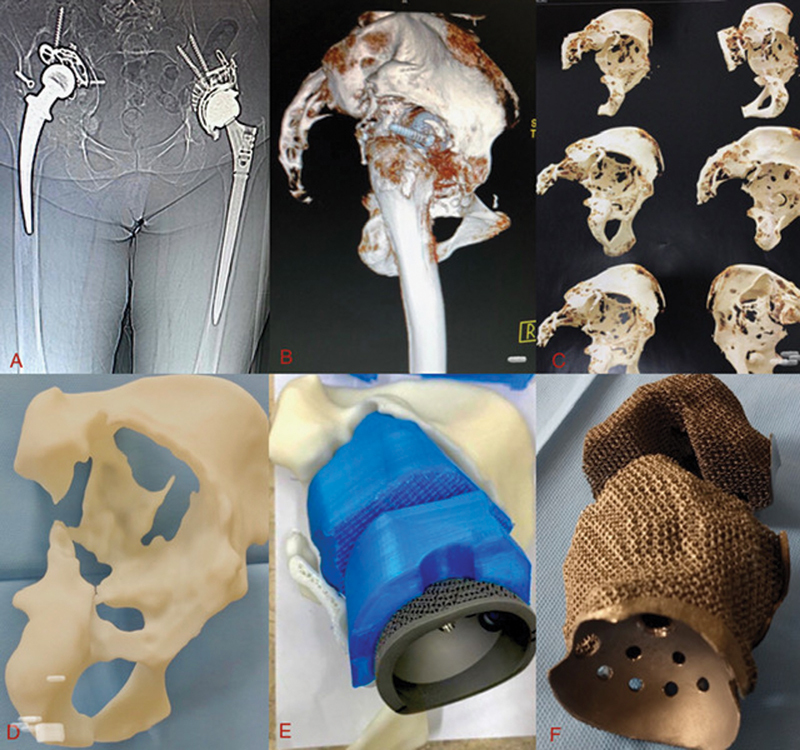
( **A** ) Computed tomography; ( **B** ) materialize; ( **C** ) segregation of the desired part; ( **D** ) 3D printing of the remaining bone; ( **E** ) 3D printing of the new component – acetabular component; ( **F** ) Customized acetabular component.

**
1 ^st^ - Documentation for authorization of the surgical procedure requested by the Brazil National Heath Surveillance Agency (ANVISA, in the Portuguese acronym);
**
  1. Detailed medical report of the patient, dated, signed, and stamped by the physician, containing the diagnosis, imaging tests, International Classification of Diseases (ICD), patient data (Individual Taxpayer Registration Number [CPF, in the Portuguese acronym], document of identification, age), technical and surgical justification stating the reason why the patient needs to use the custom-made prosthesis;
  2. Acceptance of the written informed consent form (WICF) for exceptional use of a custom-made implant, duly filled in and signed by the physician and patient.
  3. Diagnostic imaging obtained through X-RAY and/or computed tomography (CT) scans and/or magnetic resonance imaging (MRI) scans.
  4. Custom-made product manufacturer's liability statement;
  5. Custom-made prosthesis design;
  6. Detailed procedure worksheet;
  7. Flowchart and detailed manufacturing process;
  8. Copy of the Good Manufacturing Practiced (GMP) Certificate of the Company Responsible for the Components;
  9. Clinical case studies, if any.
**
2 ^nd^ - Evaluation of computed tomography images;
**
In this initial stage, the quality of the image generated by the CT and the number of “artifacts” are verified. The fewer artifacts, the easier is the image processing and the better are the results ( Fig. 1A ).
**
3 ^rd^ - Medical image processing using the Materialise MIMICS software** (Materialise NV, Leuven, Province of Flemish Brabant, Belgium);
Image processing includes “cleaning up” the image by removing artifacts so that the desired part can be viewed clearly ( Fig. 1B ).
**
4 ^th^ - Segregation of the part to be worked on;
**
  Reduce the size of the image keeping only the part to be worked on, and then creating a 3D file.
**
5 ^th^ - 3D printing of the part to be worked on;
**
At this stage, the part to be worked on, a 1:1 scale replica of the anatomy, will be 3D printed using a polymer. With the new file and the polymer used in 3d printing, begins the designing of the component to be built with the original engineering concept. At this stage, the involvement of the surgeon is essential to define and create the new component ( Fig. 1C and 1D ).
**
6 ^th^ - 3D printing of the new component;
**
We performed 3D printing in the polymer of the segregated part, in order to assess the set, assembly, design, and visualization of the best surgical approach ( Fig. 1E ).
**
7 ^th^ - New component manufacturing through additive manufacturing;
**
Using Materialise Magics 3D Printing Software (Materialise), we prepared the standard triangulate language (STL) file to manufacture the implant through the additive manufacturing process (3D printing) using titanium alloy powder as the raw material. At this stage, the solid and trabecular surfaces are easily distinguished ( Fig. 1F ).
  The new component may have geometries that require greater assembly precision, so CNC (computer numerical command) mechanization is needed around and in the center of the machining.
8 ^th^ - Machining; 9 ^th^ - Metrology; 10 ^th^ - Cleaning; 11 ^th^ - Recording; and 12 ^th^ - Sterilization.

## Surgical procedure

Having ruled out any suspicion of infection, we opted for the total hip arthroplasty revision surgery (THARS) in just one time. The hip joint was exposed by the extended Kocher Langenbeck posterior approach. 2 The femoral component cemented by extended trochanteric osteotomy was removed, followed by the acetabular component, reinforcement ring, and bone cement with subsequent removal of debris and thorough cleaning of the cavity. We observed extensive bone loss throughout the acetabular roof, and great involvement of the posterior column of the acetabulum; the native bone was present, in small amounts, in the ilium, ischium, and pubis. The remaining anatomical accidents were identified, and careful curettage was performed until the appearance of bleeding bone to receive the new components. The modular components with support on the remaining bone were used, with the cranial component being coupled and fixed by screws in the host bone in a position previously determined in the 3D model. The modular component caudal to this was fitted and fixed with screws in the cranial component and in the remaining bone. After these steps, the standard acetabular component was fixed to the customized components in a conventional way. It is important to note that the previous site of the screws was extensively studied in the 3D model.

**Fig. 2:**
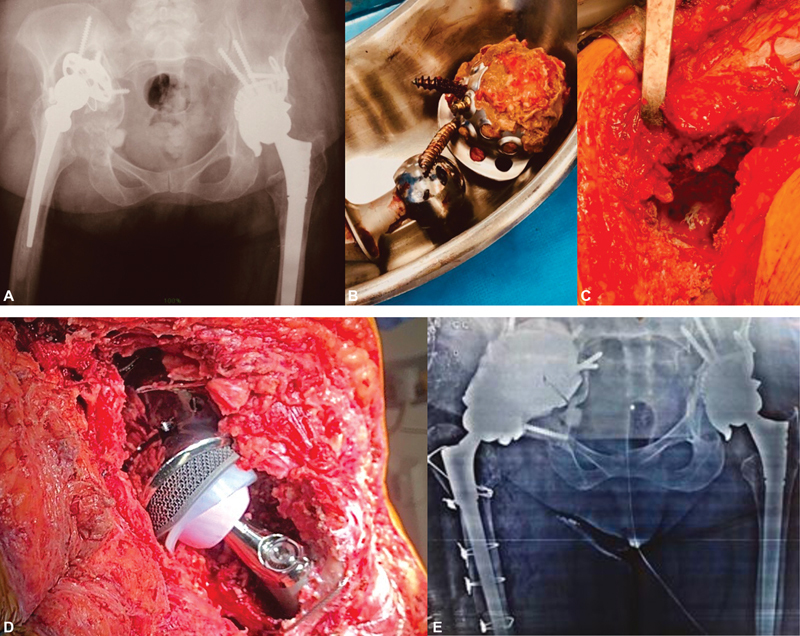
( **A** ) Preoperative radiography; ( **B** ) removed acetabular component; ( **C** ) intraoperatively; ( **D** ) intraoperative total hip arthroplasty revision surgery; ( **E** ) postoperative radiography.

The surgery was completed with the insertion of a modular femoral component, placement of the femoral head, and stability tests ( Fig. 2 ).

## Discussion

The main objectives of acetabular reconstruction surgery are to provide stability to the implant in the residual bone of the host and to reconstruct the hip biomechanics. In THARS, preoperative planning with radiographs and CT is mandatory. In case of severe bone loss, the triplanar reconstruction method should aim to evaluate the defect. 3

A variety of material options can be used to fill and repair bone defects. Greater frictional resistance and better osseointegration demonstrate the superiority of trabecular metal components and justify higher costs, especially in those patients with the most severe bone defects. 4 5 The use of customized trabecular metal prosthesis to reconstruct acetabular defects has become a viable option for correcting bone defects, because it takes into account imaging tests and the characteristics of each patient's injury. The technical difficulty involved is mainly the location of anatomical parameters, which are often lost during the osteolysis process, which is a fact observed in the reported case.

The multiple implant models are the result of integration between engineers and orthopedic surgeons to ensure the conformation of customized components and more suitable for each case, mainly the 3D models of bone defects in the pelvic and femoral anatomy. Such models can add improvements to the fixation inserting flanges to add screws in the ilium, ischium, and/or pubic bone. 6 Another advantage is to foresee the difficulties of insertion of the made-to-measure acetabular component. Ideally, these advantages lead to a shorter surgical time, management of bone defect issues, and implant stability in a separate way. The secondary stability is obtained with biological bone growth and the large surface with trabecular coating, which allows for osseointegration with long-term fixation. 7

Preoperative planning requires a lot of time. In our country, due to delays in bureaucratic procedures, preparations for the release of implants will take more time. In a study conducted by Kavalerskiy et al., the precise preoperative planning, the design, and production of implants resulted in 100% use of prototypes related to the elective surgery scheduled in an advance. This strategy allows the implant to be adjusted according to the residual bone of the host, filling the bone gap, and restoring the biomechanics of the hip. 8 9

In the present case, the osseointegration capacity was also increased, involving the use of trabecular metal made of titanium, which showed satisfactory clinical and radiological results in the mid-term follow-up study, a fact that was observed during the period of the case reported. 10 11
