# Supplementary material for: Custom-made Trabecular Metal Acetabular Component in Total Hip Revision
Source: Rev Bras Ortop (Sao Paulo). 2022 Sep 5;59(Suppl 1):e78–82. doi: 10.1055/s-0041-1735142 (PMC11254436; doi:10.1055/s-0041-1735142)
Supplement: Related texts

# Research article: Acetábulo de metal trabeculado customizado na revisão de artroplastia total do quadril

**Keywords:** acetábulo, artroplastia de quadril, impressão 3D, prótese de quadril, titânio

## Abstract

O seguinte relato de caso tem como objetivo demonstrar uma cirurgia de revisão de artroplastia total do quadril (RATQ) com o uso de componente acetabular customizado de metal trabeculado para correção de defeito acetabular severo. Atualmente, na literatura existem poucas descrições completas do planejamento e procedimentos cirúrgicos envolvendo próteses customizadas, tanto pela dificuldade técnica, inerente ao ato cirúrgico, quanto devido aos altos custos relacionados ao planejamento e materiais.

## Introdução

Nas últimas décadas, foram expostos diversos estudos que apresentam novas técnicas e materiais, com o intuito de buscar soluções plausíveis e acessíveis para a correção de falhas ósseas no quadril, com o objetivo de devolver aos pacientes uma função articular adequada. Neste estudo, apresentamos o uso de prótese de revisão acetabular customizada de metal trabeculado confeccionada de titânio em uma paciente com falha óssea acetabular Paprosky tipo 3B. 1

## Relato de caso

Paciente com 65 anos de idade, do sexo feminino, caucasiana, com histórico de doença degenerativa do quadril direito, apresentando dor e diminuição do arco de movimento. A mesma foi submetida à artroplastia total do quadril direito aos 30 anos de idade, utilizando prótese total cimentada, sem intercorrência. Após 15 anos apresentou desconforto e limitação dos movimentos do membro direito, passando por novo procedimento cirúrgico de revisão do componente acetabular com utilização de anel de reforço acetabular. Decorridos 19 anos, deste procedimento, a paciente apresentou desconforto e grave perda de amplitude dos movimentos. Após avaliação do resultado dos estudos de imagens, foi evidenciado soltura dos componentes protéticos no acetábulo e concomitantemente perda óssea acetabular, que foi classificada como Paprosky tipo 3B, sendo indicada nova abordagem.

Avaliações seriadas até o 6° mês pós-operatório demonstraram ganho do arco de movimento, osteointegração dos componentes e satisfação da paciente em relação ao resultado.

## Etapas da customização do componente acetabular

**1 ° - Documentação para autorização do procedimento cirúrgico solicitado pela Agência Nacional de Vigilância Sanitária (ANVISA);**
  1. Relatório médico detalhado do paciente, datado, assinado e carimbado pelo médico, contendo o diagnóstico, imagens, Classificação Internacional de Doenças de (CID), dados do paciente (CPF, RG, Idade), justificativa técnica e cirúrgica pelo qual o paciente necessita da utilização da prótese sob medida;
  2. Termo de responsabilidade/esclarecimento para utilização excepcional de implante sob medida devidamente preenchido e assinado pelo médico e paciente.
  3. Imagens diagnósticas obtidas através de exame de raio X e/ou tomografia computadorizada (TC) e/ou ressonância magnética (RM).
  4. Declaração de responsabilidade do fabricante de produto sob medida;
  5. Desenho da prótese sob medida;
  6. Folha de processo detalhada;
  7. Fluxograma e rota de fabricação detalhada;
  8. Cópia do Certificado de Boas Práticas de Fabricação (BPF) da empresa responsável pelo material;
  9. Estudo de caso clínico, se houver.
**2° - Recebimento e avaliação das imagens da tomografia computadorizada;**
Nesta fase inicial, verifica-se a qualidade da imagem gerada pela TC e a quantidade de “artefatos”. Quanto menos artefatos, mais fácil o tratamento e melhor resultado ( Fig. 1A ).
**3° - Tratamento da imagem com a utilização do software MIMICS – Materialise** (Materialise NV, Leuven, Privíncia de Flemish Brabant, Bélgica);
O tratamento consiste em “limpar” as imagens retirando os artefatos, assim permitindo que se tenha visão nítida da parte desejada ( Fig. 1B ).
**4° - Segregação da parte a ser trabalhada;**
  Redução da imagem mantendo apenas a parte a ser trabalhada, gerando um arquivo 3D.
**5° - Impressão 3D da parte a ser trabalhada;**
Nesta fase, a parte a ser trabalhada, réplica em escala 1:1 da anatomia, será impressa em 3D utilizando um polímero. Com o novo arquivo e a peça impressa em polímero, inicia-se o projeto do componente a ser construído com as premissas de engenharia. Nesta fase, a participação do cirurgião é fundamental para definição e criação do novo componente ( Fig. 1C e 1D ).
**6° - Impressão 3D do novo componente;**
Nesta fase é realizada a impressão 3D em polímero e da parte segregada para avaliação de conjunto, montagem, design e visualização da melhor abordagem cirúrgica ( Fig. 1E ).
**7° - Fabricação do novo componente em manufatura aditiva;**
Com a utilização do software Materialize Magics (Materialise), preparamos o arquivo de linguagem triangular padrão (STL, na sigla em inglês) para fabricar o implante pelo processo de manufatura aditiva (impressão 3D) utilizando a matéria-prima pó de liga de titânio. Nesta fase, as superfícies sólida e trabecular são distinguidas ( Fig. 1F ).
  O novo componente pode ter geometrias que necessitem de maior precisão de montagem, sendo assim, a mecanização (comando numérico computadorizado (CNC) em torno/centro de usinagem é necessária.
9° - Metrologia; 10° - Limpeza; 11° - Gravação; e 12° -Esterilização.

**Fig. 1:** ( **A** ) Tomografia computadorizada; ( **B** ) Materialise; ( **C** ) Segregação da parte desejada; ( **D** ) Impressão 3D do osso remanescente; ( **E** ) Impressão 3D do novo componente- componente acetabular; ( **F** ) Componente acetabular customizado.

## Procedimento cirúrgico

Após ter sido afatastada qualquer suspeita de infecção, foi optado pela RATQ em apenas um tempo. A articulação do quadril foi exposta pela via posterior de Kocher Langenbeck estendida. 2 Retirados o componente femoral cimentado por osteotomia trocantérica estendida, seguido do componente acetabular, do anel de reforço e cimento com subsequente remoção de debris e limpeza exaustiva da cavidade. Foi observada extensa perda óssea de todo o teto acetabular e grande comprometimento da coluna posterior do acetábulo; o osso nativo estava presente, em pequena quantidade, no ilíaco, ísquio e púbis. Foram identificados os acidentes anatômicos remanescentes, e foi realizada curetagem cuidadosa até o aparecimento de osso sangrante para receber os novos materiais. Foram utilizados componentes modulares com apoio no osso remanescente, sendo o cranial acoplado e fixo por parafusos no osso hospedeiro em posição previamente determinada no modelo 3D, e o componente modular caudal a este foi encaixado e fixado através de parafusos no componente cranial e no osso remanescente. Após estas etapas, o componente acetabular padrão foi fixado aos componentes customizados de maneira rotineira. É importante salientar que a localização prévia dos parafusos foi exaustivamente estudada no modelo 3D.

A cirurgia foi concluída com a inserção de componente femoral modular, colocação da cabeça femoral, e testes de estabilidade ( Fig. 2 ).

**Fig. 2:** ( **A** ) Radiografia pré-operatória; ( **B** ) componente acetabular retirado; ( **C** ) intraoperatório; ( **D** ) revisão de artroplastia total do quadril intraoperatória; ( **E** ) Radiografia pós-operatória.

## Discussão

Os principais objetivos da reconstrução acetabular são proporcionar estabilidade ao implante no osso residual do hospedeiro e reconstruir a biomecânica do quadril. Na RATQ, o planejamento pré-operatório com radiografias e tomografia computadorizada é obrigatório. Em caso de perda óssea grave, o método de reconstrução triplanar deve visar a dimensionar o defeito. 3

Diversas opções de materiais podem ser utilizadas para o preenchimento e reparação de falhas ósseas. A maior propriedade de resistência ao atrito e melhor osteointegração sugere a superioridade dos componentes de metal trabeculado e justifica o maior custo, principalmente naqueles pacientes com defeitos ósseos mais severos. 4 5 A reconstrução do defeito acetabular com o uso de prótese customizada de metal trabeculado surgiu como uma opção viável para a correção de defeitos ósseos, levando em consideração exames de imagem e características próprias da lesão de cada indivíduo. A dificuldade técnica envolvida se refere principalmente à localização dos parâmetros anatômicos, muitas vezes perdidos por processo de osteólise, fato este observado no caso relatado.

Os múltiplos modelos de implantes são o resultado de uma integração entre engenheiros e cirurgiões para garantir a conformação de componentes personalizados e mais adequados a cada caso, avaliando modelos 3D de defeitos ósseos na anatomia pélvica e femoral. Tais modelos podem agregar melhorias à fixação, inserindo flanges para agregar parafusos no ílio, ísquio e/ou osso púbico. 6 Outra vantagem é antecipar dificuldades de inserção do componente acetabular feito sob medida. Idealmente, essas vantagens levam a um tempo cirúrgico mais curto e ao gerenciamento dos problemas de defeitos ósseos, e à estabilidade do implante separadamente. A estabilidade secundária é obtida com o crescimento ósseo biológico e a grande superfície com revestimento trabeculado, que permite a osteointegração com fixação a longo prazo. 7

O planejamento pré-operatório requer bastante tempo e, em nosso meio, soma-se à demora do trâmite burocrático para liberação da confecção do implante. Em um estudo realizado por Kavalerskiy et al., o planejamento pré-operatório preciso, o design, e a produção de implantes levaram a uma utilização de 100% dos protótipos nos casos em relação a programação cirúrgica prévia. Essa estratégia permite ajustar o implante ao osso residual do hospedeiro, preenchendo a deficiência óssea e restaurando a biomecânica do quadril. 8 9

Neste caso, foi agregada, ainda, a capacidade de osteointegração referente ao uso de metal trabeculado produzido com titânio, que apresenta resultados clínicos e radiológicos satisfatórios em estudos com seguimento a médio prazo, fato este evidenciado no período do caso relatado. 10 11
